# Association between some environmental risk factors and attention-deficit hyperactivity disorder among children in Egypt: a case-control study

**DOI:** 10.1186/s13052-025-01843-w

**Published:** 2025-01-29

**Authors:** Riham Abdelhamid Hussein, Rania Hussein Refai, Aleya Hanafy El-zoka, Hanan Galal Azouz, Mohamed Fakhry Hussein

**Affiliations:** 1 Alexandria University, Alexandria, Egypt; 2 Bibliotheca Alexandrina, Alexandria, Egypt; 3 High Institute of Public Health, Alexandria University, Alexandria, Egypt

**Keywords:** Attention-deficit hyperactivity disorder, Egypt, Environmental risk factors, Lead, Manganese

## Abstract

**Background:**

Attention-Deficit Hyperactivity Disorder (ADHD) is a complex disease that negatively impacts the social and academic/occupational activities of children and is more common in boys than in girls.

**Methods:**

This case-control study aimed to assess the association between some environmental risk factors and ADHD among children in Alexandria, Egypt. It was carried out at the outpatient clinics of El Shatby Pediatric University Hospital in Alexandria, Egypt, with 252 children (126 cases and 126 controls). Hair samples were collected for analysis of lead and manganese levels using Atomic Absorption Spectrophotometer. A pre-designed interview questionnaire was used to determine the important environmental risk factors that may be related to ADHD.

**Results:**

Children from parents with low levels of education, living in crowded houses, and occupational exposure to chemical agents were found to be risk factors for ADHD. The mean ± SD hair lead level in ADHD children was 2.58 ± 1.95, while in controls was 1.87 ± 0.92, with a statistically significant difference (*p* < 0.001). The mean ± SD hair manganese level in ADHD children was 2.10 ± 1.54, while in controls was 1.11 ± 0.69, with a statistically significant difference (*p* < 0.001). The logistic regression model revealed that six factors had a significant association with ADHD: using of newspapers to wrap food 3 or more times a week (adjusted odds ratio (AOR) = 105.11, 95% CI: 11.18-988.26), daily TV watching by child for more than 5 h (AOR = 63.96, 95% CI: 2.56-1601.32), child’s eating commercially packed noodles 3 times or more per week (AOR = 57.73, 95% CI: 3.77–593.93), using unpackaged flour in cooking (AOR = 44.47, 95% CI: 1.83–629.80), eating sweets daily by child (AOR = 6.82, 95% CI: 1.23–37.94), and lastly elevated hair Manganese level (AOR = 3.57, 95% CI: 1.24–10.29).

**Conclusions:**

ADHD is a multi-factorial disorder, where many environmental risk factors contribute to its development. Future efforts to determine the best preventive strategy in Egypt must be based on a better knowledge of the role of environmental risk factors in the etiology of the disorder. Eliminating non-essential uses of lead and providing public education regarding the importance of safe disposal of lead-acid batteries and computers are necessary.

**Supplementary Information:**

The online version contains supplementary material available at 10.1186/s13052-025-01843-w.

## Background

Attention-deficit hyperactivity disorder (ADHD) is a persistent pattern of inattention and/or hyperactivity-impulsivity that interferes with functioning or development and has persisted for at least 6 months to a degree that is inconsistent with developmental level and that negatively impacts directly on social and academic/occupational activities [1]. It is most commonly diagnosed in children. ADHD is more common among boys than girls [2].

A meta-analysis study in 2023 reported that the worldwide prevalence of ADHD in children aged 3 to 12 years was 7.6% and in teenagers aged 12 to 18 years was 5.6% [3]. Moreover, based on parent-reported data from a U.S. health survey, the prevalence of children diagnosed with ADHD was 10.2% in 2016 [4].

In Egypt, there is no accurate data about the prevalence of ADHD among children. Its prevalence among school-age children in Fayoum City was found to be 20.5% [5]. In a cross-sectional study in Gharbia governorate, 10.5% of preschoolers were found to suffer from ADHD [6]. According to a study conducted in Egypt, ADHD is the second most common disorder [7].

Factors that contribute to the etiology of ADHD are still under investigation. ADHD is a complex disorder, with genetic and environmental risk factors contributing to its onset. There is a growing body of evidence for the involvement of environmental risk factors during pregnancy or early childhood in ADHD development. Many studies have detected the significance of environmental risk factors in children with ADHD, like prenatal exposure to heavy metals, pesticides, tobacco smoke, or unfavorable housing conditions. If these factors are controlled, the incidence of the disorder may be reduced [8–10]. Other factors including low socioeconomic status, family size, age of parents, and gender might also contribute to ADHD risk [11].

Manganese (Mn) is an essential micronutrient with low levels required for good health, but it is a potent neurotoxicant in high doses. Humans could be exposed to Mn through diverse sources; drinking water, airborne particulate matter exposure from vehicular traffic, or infant milk formula [12]. Bouchard et al. reported that a high level of Manganese in tap water was associated with increased levels of hyperactivity in children [13].

Lead (Pb) is another neurotoxin and can interfere with neurotransmitters. Bellinger et al. stated that no level of lead exposure appears to be safe in children and it is associated with various neurodevelopmental toxicities [14]. A cross-sectional study in Spain showed that exposure to secondhand tobacco smoke for more than one hour per day was associated with a higher frequency of ADHD [15]. Liu and Schelar (2012) have documented that even low levels of pesticide exposure can affect young children’s neurological and behavioral development, showing a link between pesticides and ADHD [16].

Phthalates are chemical compounds commonly found in many products such as adhesives, printing ink, and personnel care products such as baby powder, perfumes, and cosmetics. In a cross-sectional study, researchers found growing evidence that phthalate exposure may be related to ADHD [17]. Outdoor air pollution and traffic-related pollution may contribute to neurobehavioral problems in children and were found to be connected to an increased incidence of ADHD [18].

Many drugs can increase the risk of ADHD if taken during pregnancy. A study found an association between paracetamol used in pregnancy and an increased risk of ADHD or similar behavior in children [19].

Early identification and diagnosis of neuropsychiatric syndromes, such as attention-deficit hyperactivity disorder (ADHD), are crucial for optimizing treatment outcomes and minimizing long-term negative consequences. Pediatricians play a pivotal role in recognizing and addressing these conditions, as early intervention can significantly impact a child’s development, academic performance, and overall well-being. By promptly diagnosing ADHD, pediatricians can initiate appropriate interventions, such as behavioral therapy, medication management, or a combination of both, to mitigate symptoms and improve the child’s quality of life. Furthermore, early diagnosis allows for the identification of potential environmental risk factors that may contribute to ADHD, enabling targeted preventive measures and strategies to reduce the likelihood of future neuropsychiatric issues [20, 21].

Given the limited data on ADHD risk factors in Egypt, this study aimed to investigate the relationship of various environmental factors, lifestyle factors, and parental occupational exposures with the development of ADHD in children in Alexandria. Besides, we compared the levels of manganese and lead in the hair of children with and without ADHD.

## Methods

### Study setting and design

A case-control study was conducted at the outpatient clinics of El Shatby Pediatric University Hospital, Alexandria, Egypt, during the period from January 2021 till March 2022. The manuscript was carefully checked using “Strengthening the Reporting of Observational Studies in Epidemiology” (STROBE) guidelines for case-control studies to ensure that all details needed were incorporated in the manuscript [22].

### Target population

The cases were children from the outpatient behavioral pediatric clinics in El Shatby Hospital who were already diagnosed with ADHD and aged from four to sixteen years old. Controls were children without ADHD attending the other pediatric clinics in El Shatby Hospital for other causes. Both cases and controls were age- and sex- matched. Selection of cases and controls was carried out by an expert pediatrician. Exclusion criteria included physically handicapped children and children with hepatic or renal diseases.

### Sample size

A sample of two hundred fifty-two children. One hundred twenty-six per group was required to estimate the average difference of second-hand tobacco smoke metabolite (urinary cotinine) in ADHD children (geometric mean = 1.79 ng/mL, geometric SD = 3.81 ng/mL) and controls (geometric mean = 1.19 ng/mL, geometric SD = 2.81 ng/mL) using an alpha error of 0.05, which will provide a power of 80%. The sample size was calculated using Epi Info7 software [23].

### Variables

#### Outcomes

- ADHD diagnosis: Carried out by an expert according to the Diagnostic and Statistical Manual of Mental Disorders, Fifth Edition (DSM-5) criteria.

#### Exposures

- Parental occupational history and occupational hazardous exposure: These include information about parents’ occupations and any potential exposure to hazardous substances, such as heavy metals, solvents, and pesticides.
- Maternal lifestyle: These include factors such as maternal smoking, alcohol consumption, and drug use during pregnancy.
- Indoor environmental factors: These include factors such as exposure to secondhand smoke and chemical agents exposure, etc.
- Outdoor environmental factors: These include exposure to air pollution and electromagnetic fields, etc.
- Lifestyle factors of the child: These include factors such diet, and screen time, etc.
- Manganese and Lead levels: Concentrations of these elements measured in micrograms per gram (µg/g) of hair.

### Data collection methods and tools

#### Data collected from the records

Records of children who were diagnosed with ADHD and confirmed by the pediatric neurologist consultant at the university hospital, using Diagnostic and Statistical Manual of Mental Disorders 5th edition (DSM-5) were taken [1].

#### Interviewing technique

A pre-designed and pre-coded structured interviewing questionnaire (in the supplementary file under: Arabic questionnaire) was used to collect data from parents or caregivers of both children with ADHD (cases) and children without ADHD (controls). Items of the questionnaire were taken from similar, previously validated research questionnaires after an extensive review of the literature [24–26]. The questionnaire was revised by two professors (in environmental health and pediatric neurology) to ensure its validity. They suggested the addition of new items as well as the replacement and adjustment of some existing ones. The English version of the questionnaire underwent forward and backward translation by native speakers who are experts in public health.

A pilot study was carried out to pretest the questionnaire, determine its clarity, estimate the average time needed to obtain the required information (15–20 min), and identify obstacles that could be faced during the implementation of the main study. A few modifications were made to the questionnaire after the pilot study to improve its clarity.

The questionnaire included six parts. Part I of the questionnaire was about personal and socio-demographic data (name, age, age of the child’s father and mother during pregnancy, level of education of parents, job of the child’s father and mother, number of persons living in the same home, and number of rooms in the home (to determine crowding index)). Part II involved questions about medical history, obstetric history, and current pregnancy. These questions included the health status of the mother, data on the previous pregnancy (if any), the outcome of the pregnancy and neonatal problems, the birth spacing interval, problems in the antenatal and postnatal periods, problems during delivery, and the mode of delivery, in addition to data on vitamins and other drugs taken during pregnancy.

Part III contained questions about parental occupational history and occupational hazardous exposure (occupation of the parents: job title and a detailed history of the exposure to any physical (noise, high temperature, non-ionizing radiations), chemical (petroleum products, paints, or organic solvents), and biological hazards (the worker exposed to blood, infectious agents, poultry and pets)). Part IV was concerned with the lifestyle of the mother, such as tobacco smoking, maternal exposure to tobacco smoking at home, use of newspapers and magazines in food preparation (a potential source of heavy metals), and the type of cooking utensils, such as glazed ceramic pottery, for their lead content.

Part V inquired about exposure to different indoor and outdoor environmental risk factors. Indoor environmental risk factors could be drinking water problems, problems with sewage water discharge, type of fuel used, adequacy of ventilation and sun exposure, type of wall paint and floor, chemical exposure like cleaning agents, paint, or ink (sources of lead), insecticide exposure, and electromagnetic field-emitting electrical devices (e.g., mobile phone, microwave oven, computer, or television). Asking about exposure to some outdoor environmental risk factors was also carried out, such as living near highways, near excessive vehicle exhausts such as garages, bus stations, and gas stations, near industrial emissions, near agriculture areas, beside waste disposal sites, and living near mobile stations.

Part VI consisted of questions about the lifestyle of the child. Parents of the child were asked about the lifestyle of their child in the first two years of life, like chewing plastic toys (a potential source of phthalates and heavy metals), exposure to pesticides, playing in the dust or soil (a potential source of lead), exposure to severe illness that required hospitalization, exposure to electromagnetic field emitting electrical devices (e.g., mobile phone, microwave oven, computer, television), and eating habits (frequency of eating chips, fresh fruit and vegetables, soft drinks, dairy products, and fish).

#### Laboratory tests

All participants were tested for hair lead and manganese concentrations. Parents were asked to wash the child’s hair thoroughly the day before taking the sample and not put any cream on the scalp after washing. Samples of 3–4 cm in length from different areas of the scalp (close to the hair root) were collected. The extracted hair was washed in distilled water, and the samples were left to dry before weighting. Digestion by 5 ml of concentrated nitric acid was performed by the expert technician. Dilution by 5 ml of distilled water was done. A blank using 5 ml of distilled water was used for each patch of analysis for comparison.

The hair lead and manganese contents were measured using an Atomic Absorption Spectrophotometer Shimadzu model (AA-6650) at the Central Laboratory of the High Institute of Public Health, Alexandria University. The measurements were done according to the technique described in the National Institute for Occupational Safety and Health (NIOSH) Manual of Analytical Methods [27].

### Ethical considerations

The researchers sought approval from the Ethical Committee of the High Institute of Public Health, Alexandria University, Egypt for conducting the study. The researchers adhered to the International Guidelines for Research Ethics outlined in the Declaration of Helsinki for medical research involving human subjects. All subjects participated on a voluntary basis. They were informed of the study’s goals and objectives, as well as its advantages for both individuals and society. Before collecting data and samples, the participants’ parents signed a written informed consent form. Each participant’s unique code number ensured that their data was anonymous and confidential. Participants had the option to withdraw from the study at any time before its completion. Moreover, participants received assurances that all information would be used solely for research.

### Statistical design and analysis

SPSS (Statistical Package for Social Sciences) version 21 was used for data entry and analysis, and Epi-Info (Epidemiological Information Package) version 7. A result was considered statistically significant when the significance probability was less than 5% (*p* < 0.05). Frequency distribution tables, the mean, and the standard deviation were used as descriptive statistics.

The Crowding Index was calculated by dividing the number of persons by the number of rooms of the residence. The Chi–square test (χ^2^) was used for calculating significant differences between the two groups (cases and controls). While in case of more than 25% of the cells have excepted count less than 5, Fisher ‘s Exact test is used.

Regarding quantitative data, a “t-test” was used for comparing the means between the two groups for the continuous, normally distributed variables. The Mann-Whitney (U) test is used for comparison of the means of the two groups for the continuous, non-normally distributed variables. The odds ratio (OR) was calculated to assess the risk of the development of ADHD in children. A 95% confidence interval (CI) was calculated for OR. The Kruskal-Wallis (H) test was used for comparison of more than two independent variables. Models of stepwise regression analysis were also used to determine the relationship between some independent variables and ADHD development in children.

## Results

### Estimation for ADHD risk in relation to some socio-demographic characteristics, parental occupation, and parental smoking at home

The present case-control study consisted of 126 cases (89 boys and 37 girls) and 126 controls (87 boys and 39 girls). The cases had an age mean of 8.44 ± 2.27 years and the controls 8.80 ± 3.19 years. There was no significant difference between the two groups as they were matched by sex and age.

Table 1 reveals how some socio-demographic factors might be linked to ADHD. Mothers who were younger than 20 years old when they became pregnant were significantly more likely to have a child with ADHD compared to mothers who were 20 or older (OR = 17.91, 95% CI: 1.02 − 31.4) However, the age of the father did not seem to be related to the risk of ADHD.

Concerning maternal education, mothers with low educational level and those with intermediate level of education were more likely to have children with ADHD compared to highly educated mothers (OR = 12.8, 95% CI: 6.44–25.42; OR = 5.23, 95% CI: 2.4 − 11, respectively). Similarly, ADHD risk was increased by father illiteracy by 12.8 times (OR = 12.8, 95% CI: 6.35–25.85) and by 6.2 times for father with intermediate level of education (OR = 6.27, 95% CI: 3.02 − 13.01).

Parental consanguinity was considered as a risk factor for ADHD as positive consanguinity between parents elevates the odds of having ADHD children by 8 times more than negative consanguinity (OR = 8.20, 95% CI: 2.38–28.26). Furthermore, living in crowded conditions (with more than 2 people per room) was also associated with a higher risk of ADHD (OR = 7.88, 95% CI: 2.50-24.84).

Fathers who were exposed to chemical agents like petroleum products, paints, or organic solvents at work were 26 times more likely to have children with ADHD compared to fathers who were not exposed (OR = 26.09, 95% CI: 7.86–86.63).

The study found that mothers who smoked at home were more likely to have children with ADHD (OR = 2.25, 95% CI: 1.21–4.20). On the other hand, fathers smoking at home did not seem to increase the risk of ADHD in children. For more details see supplementary file tables.

**Table 1:** Estimation for risk of development of ADHD in children in relation to socio-demographic characteristics, parental occupation, and parental smoking at home (Pediatric Alexandria University Hospital, 2022)

Risk Factors		Cases (*n* = 126)	Controls (*n* = 126)	OR (95%CI)
		*N* (%)	*N* (%)	
**Age of mother at conception of the child under study (years)**				
< 20 20–35 (Ref) 35+		8(6.4) 111(88) 7(5.6)	0 (0) 117 (92.8) 9 (7.2)	17.91 (1.02–31.4) 1 0.81 (0.2–2.2)
Mean ± SD.		28.15 ± 4.6	29.33 ± 3.46	
**Age of father at conception of the child under study (years)**				
20–39 (Ref) 40+		105 (83.3) 21 (16.7)	114 (90.3) 12 (9.7)	1 1.9 (0.89–4.05)
Mean ± SD.		34 ± 5.01	33.84 ± 4.64	
**Level of education of the mother**				
Low Intermediate High (Ref)		66 (52.4) 27 (21.4) 33 (26.2)	15 (11.9) 15 (11.9) 96 (76.2)	12.80 (6.45–25.42) 5.24 (2.49–11.03) 1
**Level of education of the father**				
Low Intermediate High (Ref)		61 (48.4) 32 (25.4) 33 (26.2)	14 (11.1) 15 (11.9) 97 (77)	12.81 (6.35–25.85) 6.27 (3.02–13.01) 1
**Crowding index**				
< 1(Ref) 1- **2+**		16 (12.7) 89 (69.1) 21 (18.2)	30 (23.8) 91 (72.3) 5 (3.9)	1 1.83 (0.94–3.6) 7.88 (2.5-24.84)
**Parental consanguinity**				
No (Ref) Yes		105 (83.3) 21 (16.7)	123 (97.6) 3 (2.4)	1 8.20 (2.38–28.26)
**Mother occupation**				
Housewives (Ref) Working		104 (82.5) 22 (17.5)	96 (76.2) 30 (23.8)	1 0.677 (0.367– 1.25)
**Father occupation**				
No work (Ref) Working		3 (2.4) 123 (97.6)	2 (1.6) 124 (98.4)	1 1.544 (0.90–2.64)
**Exposure of the father to physical risk factors**				
No (Ref) Yes		120 (95.2) 6 (4.8)	125 (99.2) 1 (0.8)	1 6.25 (0.74–52.69)
**Exposure of the father to biological risk factors**				
No (Ref) Yes		118 (93.7) 8 (6.3)	123 (97.6) 3 (2.4)	1 2.78 (0.72–10.73)
**Exposure of the father to chemical risk factors**				
No (Ref) Yes		77 (61.1) 49 (38.9)	123 (97.6) 3 (2.4)	1 26.09 (7.86–86.63)
**Tobacco smoking of the mother**				
No (Ref) Yes		90 (71.4) 36 (28.6)	107 (84.9) 19 (15.1)	1 2.25 (1.21–4.20)
**Tobacco smoking of the father**				
No (Ref)		62 (49.2)	66 (52.4)	1
Yes		64 (50.8)	60 (47.6)	1.14 (0.69–1.86)

Ref is reference, OR: Odds ratio, CI: Confidence interval

### Estimation for ADHD risk in relation to the history of mother’s gestational period and the infant health status

As illustrated in Table 2, children with ADHD tended to have shorter birth intervals compared to children without ADHD. The median birth interval for children with ADHD was 4 months, while for children without ADHD it was 36 months. A longer duration of birth spaces was found to have a protective effect (OR = 0.92, 95% CI: 0.90–0.94) for children against ADHD.

Women who did not take vitamins and minerals (calcium, folic acid, and iron intake) regularly during pregnancy were more likely to have children with ADHD compared to women who did take them regularly (OR = 7.28, 95% CI: 2.10–25.28).

Concerning the delivery mode, caesarean section delivery was found to have a protective effect from ADHD (OR = 0.44, 95% CI: 0.266–0.747) as around 70% of the mothers in the controls group had Cesarean section delivery mode compared to only 51% in the cases group.

The study shows that low-birth-weight babies (less than 2500 g) were more likely to develop ADHD later in life compared to babies born at a normal birth weight (≥ 2500 g) (OR = 2.72, 95% CI: 1.59–4.68). Additionally, babies with prolonged neonatal jaundice or bronchial asthma during infancy were also at a higher risk of developing ADHD (OR = 7.44, 95% CI = 3.54–15.64; OR = 21.25, 95% CI = 2.63–171.75, respectively).

**Table 2:** Estimation for risk of development of ADHD in children in relation to the history of mother’s gestational period and the infant health status (Pediatric Alexandria University Hospital, 2022)

Risk Factors		Cases (*n* = 126)	Controls (*n* = 126)	OR (95%CI)
		*N* (%) (*n* = 73)	*N* (%) (*n* = 66)	
**Spacing between the 2 pregnancies in months**				
Min. – Max. Median (IQR)		2.0–63.0 4.0 (3.0–7.0)	3.0–108.0 36.0 (24.0–60.0)	0.92 (0.90–0.94)
**Vitamins intake**				
No		19 (15.1)	3 (2.4)	7.28 (2.10–25.28)
Yes (Ref)		107 (84.9)	123 (97.6)	1
**Delivery mode**				
Normal vaginal delivery (Ref) Caesarian Section		62 (49.2) 64 (50.8)	38 (30.2) 88 (69.8)	1 0.45 (0.27–0.75)
**Weight of the child at birth**				
Normal weight 2500 gram (Ref) Weight less than 2500 gram		68 (38.1) 58 (20.6)	96 (76.2) 30 (23.8)	1 2.72 (1.59–4.68)
**Suffering of the child from any disease**				
No diseases (Ref) Neonatal jaundice for long period Asthma		74 (58.8) 42 (33.3) 10 (7.9)	113 (89.7) 12 (9.5) 1 (0.8)	1 7.44 (3.54–15.64) 21.25 (2.63-171.75)
**Type of Feeding**				
Bottle feeding Breast feeding (Ref)		10 (7.9) 116 (92.1)	3 (2.4) 123 (97.6)	3.53 (0.95–13.16) 1

Ref is reference, OR: Odds ratio, CI: Confidence interval

### Risk estimation of ADHD regarding some maternal habits, maternal exposure to electromagnetic radiation, and exposure to some indoor and outdoor environmental factors

Table 3 shows how certain indoor habits of mothers might be linked to ADHD in their children. Families who used newspapers to wrap food at least once or twice a week were 11 times more likely to have a child with ADHD compared to families who never used newspapers (OR = 11.47, 95%CI: 4.47–29.47). Additionally, cooking with aluminum utensils frequently was also associated with a higher risk of ADHD (OR = 7.74, 95% CI: 2.24–26.76).

Using unpackaged flour to prepare food 1–2 times a week or more was associated with a significant higher odds of ADHD in children compared to using packaged flour. The more often unpackaged flour was used, the higher the estimated risk (OR = 45.97 and OR = 74.26).

Mothers who used cell phones or watching TV during pregnancy did not have a significantly higher risk of having a child with ADHD. Interestingly, mothers who used computers for more than 5 h a day during pregnancy were more common among the control group than among the ADHD group.

**Table 3:** Risk estimation of ADHD regarding some maternal habits and maternal exposure to electromagnetic radiation (Pediatric Alexandria University Hospital, 2022)

Risk Factors	Case (*n* = 126)	Control (*n* = 126)	OR (95%CI)
	No. (%)	No. (%)	
**Using of newspaper to wrap food**			
No (Ref) 1–2 times weekly ≥ 3 times weekly	48 (38.1) 28 (22.2) 50 (39.7)	118 93.7) 6 (4.8) 2 (1.5)	1 11.47 (4.47–29.47) 61.46 (14.37–262.69)
**Using Aluminum utensils in cooking**			
Once or twice per week (Ref) ≥ 3 times weekly	3 (2.4) 123 (97.6)	20 (15.9) 106 (84.1)	1 7.74 (2.24–26.76)
**Using of unpackaged flour**			
No (Ref) 1–2 times weekly ≥ 3 times weekly	46 (36.5) 52 (41.3) 28 (22.2)	122 (96.8) 3 (2.4) 1 (0.8)	1 45.97 (13.68–154.5) 74.26 (9.82–561.64)
**Daily usage of mobile phone** **during pregnancy period**			
No (Ref) < 10 min 10–30 min > 30 min	8 (6.3) 55 (43.7) 59 (46.8) 4 (3.2)	3 (2.4) 61 (48.4) 58 (46.0) 4 (3.2)	1 0.34 (0.09–1.34) 0.38 (0.10–1.51) 0.38 (0.06–2.56)
**Maternal daily usage of computer during pregnancy period**			
No (Ref) < 2 h daily 2–5 h daily > 5 h	96 (76.2) 15 (11.9) 11 (8.7) 4 (3.2)	8 (6.3) 70 (55.6) 9 (7.1) 39 (31.0)	1 0.02 (0.01–0.04) 0.10 (0.03–0.32) 0.01 (0.00–0.03)
**Maternal daily hours of TV watching during pregnancy period**			
No (Ref) < 2 h 2–5 h > 5 h	2 (1.6) 41 (32.5) 52 (41.3) 31 (24.6)	1 (0.8) 61 (48.4) 56 (44.4) 8 (6.3)	1 0.34 (0.03–3.83) 0.46 (0.04–5.27) 1.94 (0.16–24.16)

Ref is reference, OR: Odds ratio, CI: Confidence interval

### Risk estimation of ADHD regarding some maternal habits, maternal exposure to electromagnetic radiation, and exposure to some indoor and outdoor environmental factors

Table 4 shows that using household pesticides 3 or more times a week was linked to a higher odds of ADHD in children (OR = 10.89, 95%CI: 2.11–56.19). Using untreated groundwater was associated with a higher odds of ADHD compared to using treated water (OR = 6.85, 95% CI: 1.50–31.30). Additionally, having old lead pipes in the house was also linked to a higher risk of ADHD compared to using new plastic pipes (OR = 2.73, 95% CI: 1.61–4.63). Using fuels like gas cylinders or biofuels instead of natural gas was strongly associated with an increased risk of ADHD (OR = 43.46, 95% CI: 20.72–91.18).

Children who were not exposed to sun and good aeration at home were more likely to increase the odds for ADHD (OR = 2.55, 95% CI: 1.54–4.24). Living in a house with old oil paint on the walls elevated the odds for ADHD (OR = 7.36, 95%CI = 2.95–18.36), while new oil paint and plastic paints showed a protective impact (OR = 0.07, 95% CI: 0.02–0.28; OR = 0.06, 95%CI: 0.02–0.17, respectively). Families who lived near busy roads, gas stations, landfills, industrial areas, or farms were more likely to have children with ADHD.

**Table 4:** Risk estimation of ADHD regarding exposure to some indoor and outdoor environmental factors (Pediatric Alexandria University Hospital, 2022)

Risk Factors	Case (*n* = 126)	Control (*n* = 126)	OR (95%CI)
	No. (%)	No. (%)	
**Using of pesticides at home**			
No (Ref) Sometimes ≥ 3 times weekly	2 (1.6) 75 (59.5) 49 (38.9)	8 (6.3) 100 (79.4) 18 (14.3)	1 3.00 (0.62–14.54) 10.89 (2.11–56.19)
**Source of water at home**			
Water company (Ref) Groundwater untreated	114 (90.5) 12 (9.5)	124 (98.4) 2 (1.6)	1 6.85 (1.50–31.30)
**Type of pipes of water at home**			
Old leaded pipes New plastic pipes (Ref)	62 (49.2) 64 (50.8)	33 (26.2) 93 (73.8)	2.73 (1.61–4.63) 1
**Type of fuel used at home**	21 (16.7)	113 (89.7)	1
Natural gas (Ref) Others	105 (83.3)	13 (10.3)	43.46 (20.72–91.18)
**Sufficient natural lighting and aeration at home**			
No Yes (Ref)	77 (61.1) 49 (38.9)	48 (38.1) 78 (61.9)	2.55 (1.54–4.24) –
**Type of painted surfaces**			
Wallpaper (Ref) Old oil paint New oil paint Plastic paint	25 (19.8 92 (73.0) 3 (2.4) 6 (4.8)	18 (14.3) 9 (7.1) 29 (23.0) 70 (55.6)	1 7.36 (2.95–18.36) 0.07 (0.02–0.28) 0.06 (0.02–0.17)
**Living near highway**			
No (Ref) Yes	63 (50.0) 63 (50.0)	123 (97.6) 3 (2.4)	1 41.00 (12.38–135.77)
**Living near high traffic street**			
No (Ref) Yes	62 (49.2) 64 (50.8)	82 (65.0) 44 (35.0)	- 1.90 (1.17–3.28)
**Living near gas station**			
No (Ref) Yes	56 (44.4) 70 (55.6)	120 (95.2) 6 (4.8)	1 25 (10.25–61.00)
**Living near landfill**			
No (Ref) Yes	115 (91.3) 11 (8.7)	125 (99.2) 1 (0.8)	1 11.96 (1.52–94.07)
**Living near industrial area**			
No (Ref) Yes	107 (84.9) 19 (15.1)	117 (92.9) 9 (7.1)	1 2.3 (1.00 –5.32)
**Living near agriculture area**			
No (Ref) Yes	72 (57.1) 54 (42.9)	124 (98.4) 2 (1.6)	1 46.50 (11.01–196.44)

Ref is reference, OR: Odds ratio, CI: Confidence interval

### Risk estimation of ADHD in relation to lifestyle of the child, exposure to electromagnetic radiation, and dietary and snacking habits

Table 5 demonstrates how a child’s lifestyle might be linked to ADHD. Children who frequently played with or chewed plastic toys were 9 times more likely to suffer from ADHD compared to children who didn’t (OR = 9.02, 95%CI: 2.03–40.10). Children who were exposed to pesticides had 62 times higher odds of suffering from ADHD compared to those who were not exposed (OR = 62.73, 95%CI: 28.33–138.87).

The present study found that spending a lot of time in front of a television, computer, or mobile phone was linked to higher odds of ADHD. Children spending more than five hours daily in front of the TV had higher odds of ADHD (OR = 32, 95%CI: 3.22–327.77). Similarly, children using mobile phones or computers for more than two hours per day were more likely to develop ADHD (OR = 2.61, 95%CI: 1.31–5.19; OR = 2.17, 95%CI: 1.16–4.05, respectively).

Children who ate certain types of junk food were more likely to have ADHD. For example, children who ate potato chips 2–5 times a week were 27 times more likely to have ADHD (OR = 27.08, 95%CI: 12.30–59.63). Children eating commercially packed noodles daily 2 to 5 times weekly or once weekly were more susceptible to developing ADHD (OR = 39.44, 95% CI: 8.49–183.24; OR = 33.61, 95% CI: 13.00–86.88; OR = 17.09, 95% CI: 6.50–45.0, respectively). Similarly, children who drank carbonated beverages or ate sweets frequently were also at a higher risk of ADHD (OR = 3.89 and 4.0, respectively).

Children who did not drink milk at all or only once weekly had higher odds of ADHD development (OR = 21.88, 95% CI: 6.79–70.48; OR = 9.77, 95% CI: 4.14–23.06, respectively). Eating fruits and vegetables once daily or at least 1–3 times per week showed a protective role against ADHD (OR = 0.22, 95% CI: 0.13–0.38; OR = 0.12, 95% CI: 0.02–0.57, respectively).

**Table 5:** Risk of ADHD in relation to lifestyle of the child, exposure to electromagnetic radiation, and dietary and snacking habits (Pediatric Alexandria University Hospital, 2022)

Risk factors	Case (*n* = 126)	Control (*n* = 126)	OR (95%CI)
	No. (%)	No. (%).	
**Child plays with plastic toys**			
No (Ref) Yes	2 (1.6) 124 (98.4)	16 (12.7) 110 (87.3)	1 9.02 (2.03–40.10)
**Exposure of the child to household pesticides**			
No (Ref) Yes	11 (8.7) 115 (91.3)	108 (85.7) 18 (14.3)	1 62.73 (28.33–138.87)
**The child’s daily watching of TV**			
No (Ref) < 2 h 2–5 h > 5 h	1 (0.8) 16 (12.7) 44 (34.9) 65 (51.6)	4 (3.2) 52 (41.3) 62 (49.2) 8 (6.3)	1 1.23 (0.13–11.82) 2.84 (0.31–26.27) 32.50 (3.22–327.77)
**The child’s daily usage of mobile phone**			
≤ 2 h/ day (Ref) >2 h/ day	14 (11.1) 112 (88.9)	31 (24.6) 95 (75.4)	1 2.61 (1.31–5.19)
**The child’s daily usage of computer**			
≤ 2 h/ day (Ref) > 2 h/ day	19 (15.1) 107 (84.9)	35 (27.8) 91 (72.2)	1 2.17 (1.16– 4.05)
**Eating potato crisps**			
No (Ref) Once weekly 2–5 times weekly	13 (10.3) 18 (14.3) 95 (75.4)	63 (50.0) 46 (36.5) 17 (13.5)	1 1.90 (0.85–4.26) 27.08 (12.30–59.63)
**Eating commercial packed noodles**			
No (Ref) Once a week 2–5 times weekly Daily	6 (4.8) 39 (31.0) 71 (56.3) 10 (7.9)	71 (56.3) 27 (21.4) 25 (19.8) 3 (2.4)	1 17.09 (6.50–45.0) 33.61 (13.00–86.88) 39.44 (8.49–183.24)
**Drinking of carbonated beverages**			
No (Ref) Once per week 2–5 times weekly Daily	20 (15.9) 57 (45.2) 47 (37.3) 2 (1.6)	48 (38.1) 46 (36.5) 29 (23.0) 3 (2.4)	1 2.97 (1.55–5.69) 3.89 (1.94–7.81) 1.60 (0.25–10.32)
**Eating sweets**			
No (Ref)	0 (0.0)	2 (1.6)	1
2–5 times weekly Once weekly Daily	33 (26.2) 18 (14.3) 75 (59.5)	45 (35.7) 34 (27.0) 45 (35.7)	1.76 (0.74–4.18) 2.68 (0.12–58.83) 4.00 (1.75–9.13)
**Drinking of milk**			
No Once a week 2–5 times weekly Daily (Ref)	35 (27.8) 43 (34.1) 30 (23.8) 18 (14.3)	4 (3.2) 11 (8.7) 66 (52.4) 45 (35.7)	21.88 (6.79–70.48) 9.77 (4.14–23.06) 1.14 (0.57–2.28) 1
**Eating vegetable and fruits**			
No (Ref) ≤ 3 times weekly Daily	87 (69.0) 2 (1.6) 37 (29.4)	40 (31.8) 8 (6.3) 78 (61.9)	1 0.12 (0.02–0.57) 0.22 (0.13–0.38)

Ref: reference, OR: Odds ratio, CI: Confidence interval

### Assessment of lead and manganese in the hair

Table 6 shows the distribution of cases and controls according to lead and manganese concentrations in the hair of studied subjects. A higher mean of lead concentration in the hair of the cases (2.58 ± 1.95 µg/ gram) compared to controls (1.87 ± 0.92 µg/ gram) and the difference was statistically significant (*p* = 0.004), and a higher mean of manganese concentration in the hair of the cases group (2.10 ± 1.54 µg/ gram) compared to the controls group (1.11 ± 0.69 µg/ gram), and the difference was also significant (*p* < 0.001).

Higher levels of lead and manganese concentrations in children’s hair were associated with increased odds of having ADHD than low heavy metal levels (OR = 1.41, 95% CI: 1.16–1.70) and (OR = 2.26, 95% CI: 1.69–3.02).

**Table 6:** Level of lead and manganese concentration (µg/ gram) in the hair of the studied groups (Pediatric Alexandria University Hospital, 2022)

Metals	Cases (*n* = 126)	Controls (*n* = 126)	Test of significance (*p*-value)	OR (95%CI)
**Lead** (**µg/ gram)**				
Min. – Max.	0.0–13.0	0.0–4.10	U = 6274.0^*^ 0.004*	1.41 (1.16–1.70)
Mean ± SD	2.58 ± 1.95	1.87 ± 0.92		
Median (IQR)	2.20 (1.20–3.50)	1.75 (1.20–2.70)		
**Manganese (µg/ gram)**				2.26 (1.69–3.02)
Min. – Max.	0.0–7.0	0.10–2.60	U = 4870.5^*^ < 0.001*	
Mean ± SD	2.10 ± 1.54	1.11 ± 0.69		
Median (IQR)	1.85 (1.0–2.90)	1.0 (0.40–1.70)		

U: Mann Whitney test, OR: Odds ratio, CI: Confidence interval, *: Statistically significant at *p* ≤ 0.05

### Multivariate logistic regression analysis for factors affecting children with ADHD

A multivariate stepwise logistic regression model was built to determine which of the previously mentioned factors really contribute to predicting the development of ADHD. As shown in Table 7, six factors were found to be significantly associated with ADHD. The strongest factor was using newspapers to wrap food frequently (adjusted odds ratio (AOR) = 105.11), followed by watching TV for more than 5 h a day (AOR = 63.96), eating commercially packed noodles 3 or more times a week (AOR = 57.73), using unpackaged flour in cooking (AOR = 44.47), eating sweets daily (AOR = 6.82), and lastly having elevated levels of manganese in hair (AOR = 3.57).

**Table 7:** Multivariate logistic regression analysis for factors affecting children with ADHD (Pediatric Alexandria University Hospital, 2022)

Independent variables	Adjusted Odds ratio (AOR)	95% CI (LL-UL)	*p*-value
Age of the mother (< 20 y)	1.011	0.004–255.71	0.997
Mother with low level of education	1.29	0.16–10.40	0.81
Tobacco smoking of the mother (yes)	11.81	0.79–175.91	0.07
Exposure of the father to chemical risk factors (yes)	2.99	0.11–79.87	0.51
Using of unpackaged flour (yes)	44.47	1.83–629.80	0.032*
Using of newspaper to wrap food 3 or more times a week	105.11	11.18–988.26	0.001*
Living near high traffic street (yes)	7.77	0.69–87.12	0.096
The child’s daily watching of TV for more than 5 h	63.96	2.56–1601.32	0.011*
The child’s eating commercially packed noodles ≥ 3 times a week	57.73	3.77–593.93	0.008*
Eating sweets daily by child	6.82	1.23–37.94	0.028*
Elevated hair Lead level	0.58	0.21–1.61	0.29
Elevated hair Manganese level	3.57	1.24–10.29	0.018*

OR: Odds ratio, CI: Confidence interval, LL: Lower limit, UL: Upper Limit, *: Statistically significant at *p* ≤ 0.05

## Discussion

This study aimed to investigate the association between various environmental factors, lifestyle factors, and parental exposures and the development of ADHD in children. Our findings suggest that several factors, including exposure to environmental pollutants, unhealthy lifestyle choices, parental occupational exposures, and certain health conditions, are linked to an increased risk of ADHD.

The present study showed that mothers who were younger than 20 years old were more likely to have children with ADHD. This finding was supported by other research that has shown a link between younger mothers and ADHD. Younger mothers may be less prepared to raise children, have fewer resources, and face more challenges like financial problems compared to older mothers [28].

The current study found that parents with lower levels of education were more likely to have children with ADHD. Similarly, other studies reported that fathers with low education were more likely to have children with ADHD. High paternal education may lead to better living conditions and an increase in understanding of children’s needs and their mental health [29, 30].

Living in crowded conditions (with more than 2 people per room) was associated with a higher risk of ADHD. This was in accordance with a study carried out by Sengupta et al. that has found a link between crowded living conditions and ADHD. This could be explained as overcrowding conditions enhance family problems and physical violence, which could provoke ADHD [31]. Positive consanguinity increased the risk of ADHD development in children by eight times. Soliman et al. (2023) reported that the presence of consanguinity between parents was a risk factor for ADHD [32].

Fathers who worked with chemicals like petroleum products, paints, and organic solvents were more likely to have children with ADHD. This finding was in line with an Egyptian study which found that all workers in a gasoline station were exposed to higher concentrations of benzene, toluene, and ethyl benzene, above the permissible limits of the American Conference of Governmental Industrial Hygienists. Workers at a gasoline station exposed to benzene via inhalation had an increasing DNA fragmentation and higher frequency of micronuclei in their blood samples; this is due to the genotoxic effect of benzene [33]. Moreover, occupational parental exposure to benzene was associated with an increased incidence of chromosomally defective sperms as well as disruption in the offspring brain neurotransmitters [34].

Regarding birth spacing, current study findings suggested a protective effect of longer birth spacing for ADHD in children. This is consistent with a Finnish study that justified that short birth spacing can be related to depletion of maternal nutrients, especially folate and polyunsaturated fatty acids, which are linked to neurodevelopmental adverse outcomes [35].

Women who did not take vitamins and minerals regularly during pregnancy were more likely to have children with ADHD. This could be explained by the absence of micronutrients such as folate, which is involved in various pathways essential for the growth of children due to its role in DNA synthesis and central nervous system maturation [36]. In addition, maternal anemia, especially in early pregnancy, was associated with an increased risk for ADHD development, as iron is necessary for several developmental processes and for the synthesis of neurotransmitters that are implicated in the etiology of ADHD [37].

Our study revealed that Cesarean Section delivery could be protective against having a child with ADHD. This result was inconsistent with other studies that found an association between Caesarian Section delivery and an increased risk of ADHD [38, 39]. The reason for these conflicting findings may be due to other factors that can affect the risk of ADHD, such as unexpected complications during vaginal delivery that can lead to a lack of oxygen for the baby (hypoxia). Hypoxia can harm brain development and increase the risk of disorders like ADHD [40].

In the present study, babies born with low birth weight (less than 2,500 g) were more likely to develop ADHD later in life. This result matches a meta-analysis that stated that low birth weight (less than 2500 g) or less than 37 weeks of gestational age was associated with ADHD. Low birth weight causes hypothalamic-pituitary-adrenal axis dysregulation and perinatal systemic inflammation that can cause structural and functional brain disorders such as ADHD and other psychiatric and developmental disorders [41].

The current study found that babies with jaundice were more likely to develop ADHD. This is similar to another research that has shown a link between jaundice and ADHD. It stated that high serum bilirubin concentrations may precipitate in neurons and induce neurological damage that might cause dysfunction of the frontostriatal network [42].

The findings of our study showed that children suffering from bronchial asthma had elevated odds of having ADHD compared with non-asthmatic children. This result was matched with a systematic review and meta-analysis study that justified a significant association between bronchial asthma and ADHD [43].

In our study, smoking mothers had higher odds of having ADHD children than non-smoking mothers. This was in accordance with a study carried out by Minatoya et al. (2019) which reported that maternal smoking may contribute to the increased risk of ADHD in offspring [44]. This can be explained as nicotine is one of the toxic substances that cigarettes contain and can cross the placenta to reach the foetus. Nicotine can affect the fetal central nervous system, creating neurological insults during the formation of the brain of the fetus and increasing the chance of having ADHD in early childhood [45].

Our data showed that breastfeeding for a period of 6 months to 1 year was protective against ADHD. These findings may be due to the neurodevelopmental benefit of breast milk and its beneficial concentration of essential fatty acids that play an important role in normal brain development, such as Omega-6 arachidonic acid, the Omega-3 eicosapentaenoic acid (EPA), and the Omega- 3 docosahexaenoic acid (DHA) [46].

Regarding the impact of some indoor risk factors on the development of ADHD, food made of unpackaged flour or packaging food in newspapers increased ADHD risk than non-users of these kinds as they may represent possible sources of exposure to lead [47]. Furthermore, ADHD children had higher odds of cooking food with aluminum utensils than the controls group. Children exposed to aluminum during their early childhood are often left with permanent neurological sequelae that include attention deficits and behavioral reactivity [48].

Our data showed that domestic consumption of untreated groundwater elevated the odds of having children with ADHD compared to using treated water. Untreated groundwater may contain a high concentration of Mn (Manganese). This is in accordance with a nationwide cohort study that showed an association between increasing levels of Mn in groundwater and the risk of ADHD [49].

In the present study, water from old pipes was shown to present a risk for ADHD. The Centers for Disease Control and Prevention (CDC) stated that the most common sources of lead in drinking water are lead pipes and plumbing fixtures. Lead could disrupt the dopaminergic system in the developing brain of a child, leading to the appearance of ADHD manifestations [50].

Our findings showed that unexposure to the sun and having bad aeration at home increased the odds of ADHD in the children compared to those exposed to sufficient sun and good aeration. A study that assessed the relationship between the prevalence of ADHD and solar intensity reported a lower prevalence of ADHD in areas with good sun exposure [51].

According to the present research, children who lived near highways, gas stations, landfills, industrial areas, or agricultural areas had elevated odds of ADHD compared to those who lived away from these areas. Living near a high-traffic street or highway increases exposure to traffic air pollutants, such as polycyclic aromatic hydrocarbons and nitrogen dioxide, which are associated with ADHD development. Children living near a high-traffic street are more exposed to higher concentrations of Particulate Matter 10 micrometers or less in diameter (PM_10_) and nitrogen dioxide, which were associated with a higher incidence of ADHD in childhood [52, 53].

A child’s residence near an agricultural area can be associated with ADHD as he could be exposed to pesticides [54]. A study carried out by Saez et al. (2018), found that living near an agricultural area, a highway, or an industrial area was associated with an increased risk of ADHD [55].

Our study showed that spending long hours in front of a screen, whether a television, computer, or mobile phone, has been significantly associated with higher odds of ADHD. The statistically significant difference between cases and controls regarding exposure of children to electromagnetic radiation was in accordance with other studies that stated that prolonged screen time was associated with a higher risk of ADHD [56, 57].

Early childhood experiences can be overstimulated by television, mobile devices, or computers, which may result in ADHD. Children who spend a lot of time in front of screens are more exposed to rapid visual changes at crucial stages of brain development, which will cause a lot of brain stimulation. Additionally, watching TV and videos, especially before the age of two, will have an impact on a child’s language and short-term memory development as well as their sleep and concentration patterns [58].

Frequent exposure to household pesticides elevated the odds of having ADHD compared to those not exposed. This finding was matched with a study that stated that urinary 3- phenoxybenzoic acid (which is a metabolite used to assess exposure to pyrethroid pesticides, the most commonly used household pesticide) was found to be above the permissible limit in the ADHD children [59]. The result was also in line with a review study, which suggested that children’s low-level pesticide exposure may be associated with ADHD [60]. This can be justified as household pesticides alter the dopamine system and alter D1 dopamine receptor levels, causing hyperactivity, attention deficits, and impulsive-like behavior in children [61].

Current study findings illustrated a risk of ADHD regarding playing with and chewing plastic toys. Plastic toys could contain bisphenol A (BPA) or phthalates that may affect the neurodevelopment of the child. A case-control study supported this idea and stated that BPA exposure was associated with negative behavioural and cognitive consequences [62]. A literature review reported that the hypothalamic-pituitary-gonadal, adrenal, and thyroid axis, which is essential for the process of neurodevelopment, is dysregulated by phthalates. Phthalates disrupt intracellular nuclear receptors that affect brain processes, which could lead to various neurological diseases, including ADHD [63].

As for the impact of some unhealthy food habits, the results of our study were in accordance with Del-Ponte et al. (2019), as they stated in a systemic review and meta-analysis study that junk foods were considered a risk factor for ADHD [64]. Regarding milk as well as fruits and vegetables consumption, our finding was similar to that of Wang et al. (2019), who found that vitamin D deficiency was higher in ADHD children compared to healthy children and indicated deficiency in consumption of milk and fresh food [65].

The present study reported that high consumption of sweets and carbonated beverages elevated the odds of suffering from ADHD compared to non-consumers. This result was in accordance with Paglia et al. (2019), who stated that high concentrations of sugar found in sweets and soft drinks can cause hyperactivity in children when taken in large quantities [66].

Lead is a potent neurotoxicant and can interfere with neurotransmitters, especially in the developing brain. Lead could be found in tobacco smoking, old painted walls, old water pipes, and air pollution from the combustion of gasoline containing tetraethyl lead or traffic densities [67]. The mean hair lead level in the current study was 2.58 ± 1.95 µg/ gram for ADHD children, which was significantly higher than controls. This was in accordance with a case-control study that reported a strong relationship between ADHD children and blood lead level [68].

According to the Mn level in hair, there is no globally accepted universal reference range and a cutoff point for manganese toxicity [69]. Menezes-Fihlo et al. (2009) used a cutoff point for hair Mn of 1.2 µg/gram [70], while O’Neal and Zheng (2015) determined that the normal Mn level in hair was ≤ 2.6 µg/gram [71].

In the current study, the mean hair Mn level in cases was about double the value for controls (2.10 ± 1.54 µg/gram vs. 1.11 ± 0.69 µg/gram). The current findings agree with the results of Shin et al. (2015), where high levels of Mn were also found in the hair of ADHD children [72]. A high Mn level in ADHD is an indication of the environmental pollution that children are exposed to, either from untreated drinking water or from air pollution. Mn could affect the brain neurotransmitters and the dopaminergic system that are involved in ADHD etiology [49].

### Limitations and strengths

There are some limitations of the study. First, some errors can be expected due to the risk of recall bias and misclassification mistakes, which are found in any case-control study since the majority of exposure data relies on self-reported history. The study design also made it difficult to be sure of certain causes of the disease and difficult to control for all potential confounders. Second, because participants lacked genetic information, it was impossible to assess how genetic variation would affect the link between risk variables and ADHD risk. Third, humans are rarely exposed to a single agent over time, so the complexity of exposures makes it hard to catch the main causes as some factors may augment the effects of others.

The current research has many points of strength. First, it studied many environmental risk factors that could be omitted by previous studies, and it matched the cases and controls on some confounding factors. Second, it assessed the risk factors through a questionnaire and lab tests. The researchers performed tests for lead and manganese in the hair of the participants, which are indicators of chronic exposure to environmental pollution. Third, all significant risk factors in bivariate analysis were introduced into the logistic regression model to assess which factors had the most important effect on ADHD.

## Conclusion

ADHD is a complex condition influenced by many factors, including environmental factors. The present study found that several environmental risk factors might increase the risk of ADHD in Egyptian children. These factors include using newspapers to wrap food, spending too much time on screens, eating certain processed foods, eating too many sweets, using unpackaged flour in cooking, and having high levels of heavy metals. Future research and efforts to prevent ADHD should focus on reducing exposure to these factors and encouraging healthy life habits.

## Electronic supplementary material

Below is the link to the electronic supplementary material.

Supplementary Material 1

Supplementary Material 2

Supplementary Material 3

Supplementary Material 4

## Data Availability

The datasets used and/or analyzed during the current study are available from the corresponding author upon reasonable request.

## Abbreviations

ADHD: Attention-Deficit Hyperactivity Disorder
AOR: Adjusted Odds Ratio
BPA: Bisphenol A
CDC: the Centers for Disease Control and Prevention
DHA: Docosahexaenoic acid
DSM-5: Diagnostic and Statistical Manual of Mental Disorders 5th edition
EPA: Eicosapentaenoic acid
IQR: Interquartile Range
MC: Monte Carlo test
Mn: Manganese
NIOSH: National Institute for Occupational Safety and Health
OR: Odds Ratio
Pb: Lead
PM_10_: Particulate Matter 10 micrometer or less in diameter
SD: Standard Deviation
