# Supplementary material for: Association between some environmental risk factors and attention-deficit hyperactivity disorder among children in Egypt: a case-control study
Source: Ital J Pediatr. 2025 Jan 29;51:19. doi: 10.1186/s13052-025-01843-w (PMC11776284; doi:10.1186/s13052-025-01843-w)
Supplement: Supplementary file 1 — Supplementary Material 1 [file 13052_2025_1843_MOESM1_ESM.docx]

**استمارة اسئلة**

**رقم الاستمارة**

**التاريخ:**

**تنتمي الى : (1)مجموعة حالات (0) المجموعة الضابطة**

**I**

**البيانات الشخصية :**

1. **الاسم:**
2. **السن :**
3. **العنوان الحالي:**
4. **فترة الاقامة بالعنوان المذكور بالسنوات:**
5. **مكان الاقامة أثناء الحمل:**
6. **سن الام اثناء الحمل:**
7. **سن الاب اثناء الحمل:**

|  | **6- اتمت تعليم جامعي/ فوق المتوسط** | **5- اتمت تعليم ثانوي /فني** | **4- اتمت تعليم اعدادي** | **3- اتمت تعليم ابتدائي** | **2- تقرأ وتكتب** | **1- لا تقرأ او تكتب** | **مستوى تعليم الام** | **8.** |
| --- | --- | --- | --- | --- | --- | --- | --- | --- |
|  | **6-اتم تعليم جامعي/ فوق المتوسط** | **5-اتم تعليم ثانوي /فني** | **4-اتم تعليم اعدادي** | **3-اتم تعليم ابتدائي** | **2- يقرأ ويكتب** | **1-لا يقرأ او يكتب** | **مستوى تعليم الأب** | **9.** |
|  |  |  | **4-بدون اجابة** | **3- يكفي ويفيض** | **2-بالكاد** | 1. **لا** | **هل دخل الاسرة يكفي الاحتياجات؟** | **10.** |
|  |  |  |  |  |  |  | **عدد أفراد العائلة الذين يسكنون في نفس منزل الطفل** | **11.** |
|  |  |  |  |  |  |  | **عدد حجرات المنزل بدون المطبخ والحمام** | **12.** |
|  |  |  |  |  |  |  | **نسبة عدد الافراد الى عدد حجرات المنزل** | **13.** |
|  |  |  |  |  | 1. **يوجد قرابة** | **1-لا توجد** | **هل يوجد قرابة بين الاب والام** | **14.** |

**II**

**اسئلة عن الحالة الصحية وتاريخ الحمل والولادة للام :**

|  |  |  |  |  |  | **ترتيب هذه الولادة في الطفل موضع الدراسة** | **15.** |
| --- | --- | --- | --- | --- | --- | --- | --- |
|  |  |  | **(2-) لا أعرف** | **2-نعم** | **1-لا** | **هل تعرضت الام للإجهاض قبل هذا الحمل** |  |
|  |  |  |  |  |  | **هل وجدت مشاكل اثناء فترة الحمل :** | **16.** |
|  | **6- مشاكل بالتنفس** | **5-مشاكل بالرحم او انذار باجهاض** | **4-ضغط عالي** | **3-سكر حمل** | **2-انيميا الدم** | **1-لا يوجد مشاكل** |  |
|  |  |  | **(-1) لا يوجد حمل سابق** | **9- اخرى تذكر** | **8--تسمم بالدم** | **7- صفراء شديدة ولمدة طويلة :** |  |
|  |  |  |  | **(-1) لا يوجد حمل سابق** |  | **الفترة بين الحمل السابق والحمل ف الطفل موضع الدراسة بالأشهر ؟** | **17.** |
|  |  |  | **(2-) لا أعرف** | **2-نعم** | **1-لا** | **هل عانيت من ارتفاع درجة الحرارة في الاشهر الاولى للحمل في الطفل محل الدراسة ؟** | **18.** |
|  |  |  | **(2-) لا أعرف** | **2-نعم** | **1-لا** | **هل تعرضت الام لدخول مستشفى اثناء الحمل في الطفل محل الدراسة ؟** | **19.** |
|  |  |  | **(2-) لا أعرف** | **2--نعم** | **1-لا** | **هل تناولت الام مادة البارسيتامول اثناء فترة حمل الطفل موضع الدراسة؟** | **20.** |
|  |  |  | **(2-) لا أعرف** | **2-نعم** | **1-لا** | **هل تناولت الام اي ادوية الاكتئاب في بداية الحمل في الطفل موضع الدراسة؟** | **21.** |
|  |  |  | **3-اخرى تذكر** | **2-الحديد** | **1-حمض الفوليك** | **هل تناولت اي من فيتامينات اثناء الحمل؟** | **22.** |
|  |  | **(1-) لم اتناول اي فيتامينات** | **3-بعد الثلاث شهور الاولى** | **2-في الشهور الثلاثة الأولى للحمل** | **1-قبل الحمل** | **متى بدأت في تناول الفيتامينات؟** | **23** |
|  |  |  |  | **2-قيصرية** | **1-طبيعية** | **نوع الولادة في الحمل موضع الدراسة** | **24.** |
|  | **4- اخرى تذكر** | **3- مشاكل بالتنفس** | **-2 صفراء شديدة ولمدة طويلة** | **1-وزن الطفل اقل من 2500 جرام** | **0-طفل مكتمل النمو وزنه اكثر او يساوي 2500 جرام ولا يعاني من مشاكل** | **هل كان الطفل موضع الدراسة- بعد الولادة يعاني من اي من المشاكل التالية:** | **.25** |
|  |  |  |  | **2-نعم** | **1-لا** | **هل قامت الام برضاعة الطفل موضع الدراسة رضاعة طبيعية** | **26.** |
|  |  | **4- اكثر من عام** | **3-من ستة شهور لعام واحد** | **2-من 3-6 شهور** | **1-اقل من ثلاث شهور** | **في حالة نعم : مدة الرضاعة الطبيعية :** | **27.** |

**III**

**اسئلة عن العمل والتعرض اثناء العمل للعوامل البيئية (اثناء الحمل وبعد الولادة مباشر):**

|  |  |  |  |  | **ما هو عمل الام؟** | **28.** |
| --- | --- | --- | --- | --- | --- | --- |
|  |  |  |  | **2- تعمل**  **-** | **1- ربة منزل**  **-** |  |
|  |  |  |  |  | **هل تتعرض الام اثناء العمل الى اي من العوامل الفيزيائية بشكل متكرر:** | **29.** |
|  | **5-اهتزازات زائدة** | **4-اشعة غير مؤينة** | **3-اشعة مؤينة** | **2-اصابات بدنية** | **1-درجات حرارة عالية** |  |
|  |  |  |  | **(1-)لا تعمل** | **6-عوامل اخرى تذكر** |  |
|  |  |  |  |  | **هل تتعرض الام اثناء العمل الى اي من العوامل البيولوجية الاتية بشكل متكرر؟** | **30** |
|  | **5-حيوانات او طيور** | **4-طعام ملوث** | **3-ماء ملوث** | **2-مرضى بأمراض معدية** | **1-دم او مشتقاته** |  |
|  |  |  |  |  | **(1-)لا تعمل** |  |
|  |  |  |  |  | **هل تتعرض الام اثناء العمل الى اي من العوامل الكيمائية الاتية بشكل متكرر** | **31.** |
|  | **5- مشتقات بترولية** | **4- احبار واصباغ** | **3-غازات من حرق قمامة** | **2- طلاء او مذيبات طلاء** | **1-مواد تنظيف ومذيبات عضوية** |  |
|  |  |  | **(1-)لا تعمل** | **7- عوامل اخرى** | **6-ادوية وغازات طبية** |  |
|  |  |  |  |  | **ما هو عمل الاب؟** | **32.** |
|  |  |  |  | **2-لايعمل** | **1- يعمل** |  |
|  |  |  |  |  | **هل يتعرض الاب اثناء العمل الى اي من العوامل الفيزيائية بشكل متكرر** | **33.** |
|  | **5- عوامل اخرى تذكر** | **4-اشعة غير مؤينة** | **3-اشعة مؤينة** | **2-اصابات بدنية** | **1-درجات حرارة عالية** |  |
|  |  |  |  |  | **(1-)لا يعمل** |  |
|  |  |  |  |  | **هل يتعرض الاب اثناء العمل الى اي من العوامل البيولوجية الاتية بشكل متكرر؟** | **34.** |
|  | **5-حيوانات او طيور** | **4-طعام ملوث** | **3-ماء ملوث** | **2-مرضى بأمراض معدية** | **1-دم او مشتقاته** |  |
|  |  |  |  |  | **(1-)لا يعمل** |  |
|  |  |  |  |  | **هل يتعرض الاب اثناء العمل الى اي من العوامل الكيمائية الاتية بشكل متكرر** | **35.** |
|  | **5- مشتقات بترولية** | **4- احبار واصباغ** | **3-غازات من حرق قمامة** | **2- طلاء او مذيبات طلاء** | **1-مواد تنظيف ومذيبات عضوية** |  |
|  |  |  | **(1-)لا يعمل** | **7- عوامل اخرى** | **6-ادوية وغازات طبية** |  |

**IV**

**اسئلة عن نمط حياة الام والعوامل التي تعرضت لها اثناء الحمل :**

|  |  | **(2)نعم** | **(1)لا** | **تدخين الام للسجائر :** | **36.** |
| --- | --- | --- | --- | --- | --- |
|  |  | **(2)نعم** | **(1)لا** | **تدخين الاب للسجائر:** | **37.** |
|  |  | **(2)نعم** | **(1)لا** | **تدخين الام للشيشة:** | **38.** |
|  |  | **(2)نعم** | **(1)لا** | **تدخين الاب/ اخرون للشيشة:** | **39.** |
|  |  | **(2)نعم** | **(1)لا** | **تناول الام للمسكرات (الخمور او المخدرات )** | **40** |
|  |  | **(2)نعم** | **(1)لا** | **تناول الاب للمسكرات (الخمور او المخدرات )** | **41.** |
|  | **(3)اكثر من او يساوى 3 مرات اسبوعيا** | **(2)احيانا** | **(1)لا** | **استخدام ورق الجرائد/ المجلات في اعداد او حفظ الطعام ؟** | **42** |
|  | **(3)اكثر من او يساوى 3 مرات اسبوعيا** | **(2)مرتين اسبوعيا** | **(1)لا** | **استخدام اواني من الالومنيوم في الطهي؟** | **43.** |
|  |  |  |  | **تناول الام لاي من الاغذية/ المشروبات الاتية (خاصة اثناء الحمل) :** | **44** |
|  | **(3)اكثر من او يساوى 3 مرات اسبوعيا** | **(2)احيانا** | **(1)لا** | **دقيق سائب** | **45.** |
|  | **(3)اكثر من او يساوى 3 مرات اسبوعيا** | **(2)احيانا** | **(1)لا** | **اطعمة او مشروبات معلبة** | **46.** |
|  | **(3)اكثر من او يساوى 3 مرات اسبوعيا** | **(2)احيانا** | **(1)لا** | **اسماك نيلية** | **47.** |
|  | **(3)اكثر من او يساوى 3 مرات اسبوعيا** | **(2)احيانا** | **(1)لا** | **اسماك بحرية** | **48.** |

**V**

**اسئلة عن تعرض الام لبعض العوامل داخل وخارج المنزل ( اثناء الحمل وبعد الولادة مباشرة ):**

|  | **3-مياه ابار معالجة** | **2- مياه ابار غير معالجة** | **-1- شركة المياه** | **ما هو مصدر مياه الشرب بالمنزل** | **50.** |
| --- | --- | --- | --- | --- | --- |
|  | **(2-)لا نعرف** | **2- بلاستيكية حديثة** | **1- قديمة** | **مواسير المياه** | **51.** |
|  |  |  |  | **نوع الوقود المستخدم داخل المنزل ؟** | **52..** |
|  | **4- وقود اخر يذكر** | **3-خشب /فحم/روث حيوانات** | **2- انابيب البوتاجاز** | **1-غاز طبيعي** |  |
|  | **(2-)لا نعرف** | **(2)نعم** | **(1)لا** | **وجود تهوية كافية بالمنزل** | **53.** |
|  | **(2-)لا نعرف** | **(2)نعم** | **(1)لا** | **هل تدخل الشمس المنزل بطريقة كافية:** | **54.** |
|  |  |  |  | **نوع تغطية حوائط المنزل؟** | **55.** |
|  | **4- طلاء زيتي قديم** | **3-طلاء زيتي حديث** | **2-بلاستيك** | **1- ورق حائط** |  |
|  |  |  | **(2-)لا نعرف** | **5- نوع تغطية اخر** |  |
|  | **(2-)لا نعرف** | **(2)نعم** | **(1)لا** | **هل تم تغيير الاثاث او عمل تجديدات بالمنزل اثناء او بعد الولادة مباشرة للطفل موضع الدراسة ؟** | **56.** |
|  |  |  |  | **هل تستخدم الام اي من المواد الكيماوية الاتية (اثناء الحمل وبعد الولادة مباشرة )** |  |
|  | **(3)اكثر من او يساوى 3 مرات اسبوعيا** | **(2)يوميا** | **(1)لا** | **منظفات صناعية ومزيلات بقع؟** | **57.** |
|  | **(3)اكثر من او يساوى 3 مرات اسبوعيا** | **(2)احيانا** | **(1)لا** | **مذيبات عضوية (مزيل طلاء/ مذيبات صمغ)** | **58.** |
|  | **(3)اكثر من او يساوى 3 مرات اسبوعيا** | **(2)احيانا** | **(1)لا** | **مواد كيماوية** | **59.** |
|  | **(3)اكثر من او يساوى 3 مرات اسبوعيا** | **(2)احيانا** | **(1)لا** | **مبيدات حشرية** | **60.** |
|  | **(3)اكثر من او يساوى 3 مرات اسبوعيا** | **(2)احيانا** | **(1)لا** | **احبار واصباغ** | **61.** |

**مدة تعرض الام لأي من الاجهزة الكهربائية الاتية يوميا ( اثناء الحمل وبعد الولادة مباشرة)**

|  |  |  |  | **التليفون المحمول** | **62.** |
| --- | --- | --- | --- | --- | --- |
|  | **4-أكثر** | **3-من 10-30 دقيقة** | **2-اقل من عشر دقائق** | **1-لا يوجد** |  |
|  |  |  |  | **الكمبيوتر** | **63.** |
|  | **4-أكثر** | **3- من 2-5 ساعات** | **2-اقل من ساعتين** | **1-لا يوجد** |  |
|  |  |  |  | **التليفزيون** | **64.** |
|  | **4-أكثر** | **3- من 2-5 ساعات** | **2-اقل من ساعتين** | **1-لا يوجد** |  |

**هل تسكن الام او الطفل بجوار اي من الاتي (اثناء الحمل وبعد الولادة مباشرة ):**

|  | **(2-) لا نعرف** | **(2)نعم** | **(1)لا** | **طرق رئيسية او كباري ذات كثافة مرورية عالية** | **65.** |
| --- | --- | --- | --- | --- | --- |
|  | **(2-) لا نعرف** | **(2)نعم** | **(1)لا** | **جراج / موقف نقل/ محطة بنزين** | **66.** |
|  | **(2-) لا نعرف** | **(2)نعم** | **(1)لا** | **مكان لجمع وحرق القمامة** | **67.** |
|  | **(2-) لا نعرف** | **(2)نعم** | **(1)لا** | **مصانع** | **68.** |
|  | **(2-) لا نعرف** | **(2)نعم** | **(1)لا** | **حقول زراعية تستخدم فيها مبيدات حشرية** | **.69** |
|  | **(2-) لا نعرف** | **(2)نعم** | **(1)لا** | **محطة تقوية لشبكة تليفون المحمول** | **70.** |

**VI**

**نمط حياة الطفل**

|  | **(2-) لا نعرف** | **(2)نعم** | **(1)لا** | **هل يلعب الطفل بألعاب بلاستيك ويمضغها في فمه** | **71.** |
| --- | --- | --- | --- | --- | --- |
|  | **(2-) لا نعرف** | **(2)نعم** | **(1)لا** | **هل يتعرض الطفل للمبيدات داخل او خارج المنزل؟** | **72.** |
|  |  |  |  | **مدة تعرض الطفل للأجهزة التالية :** |  |
|  |  |  |  | **التليفزيون** | **73.** |
|  | **4-أكثر** | **3- من 2 إلى 5 ساعات** | **2-اقل من ساعتين** | **1-لا يوجد** |  |
|  |  |  |  | **التليفون المحمول** | **74.** |
|  | **4-أكثر** | **-3 من 10الى 30 دقيقة** | **2-اقل من عشر دقائق** | **1-لا يوجد** |  |
|  |  |  |  | **الكمبيوتر** | **75.** |
|  | **4-أكثر** | **3- من 2 إلى 5 ساعات** | **2-اقل من ساعتين** | **1-لا يوجد** |  |
|  |  |  |  | **اسئلة خاصة بتغذية الطفل :** |  |
|  |  |  |  | **هل يأكل الطفل الشيبسي؟** | **76.** |
|  | **4-يوميا** | **3 - من 2 إلى 5 مرات اسبوعيا** | **2-مرة اسبوعيا** | **1-لا** |  |
|  |  |  |  | **هل يأكل الاطفال الفاكهة الطازجة والخضروات؟** | **77.** |
|  | **4-يوميا** | **3 - من 2 إلى 5 مرات اسبوعيا** | **2-مرة اسبوعيا** | **1-لا** |  |
|  |  |  |  | **هل يشرب الطفل المياه الغازية ؟** | **78.** |
|  | **4-يوميا** | **3 - من 2 إلى 5 مرات اسبوعيا** | **2-مرة اسبوعيا** | **1-لا** |  |
|  |  |  |  | **هل يأكل الطفل منتجات الالبان او يشرب اللبن ؟** | **79.** |
|  | **4- يوميا** | **3 - من 2 إلى 5 مرات اسبوعيا** | **-2-مرة اسبوعيا** | **1- لا** |  |
|  |  |  |  | **اسماك نيلية** | **80.** |
|  |  | **(3)اكثر من او يساوي 5مرات شهريا** | **2-أسبوعيا** | **1-لا** |  |
|  |  |  |  | **هل يأكل الطفل الاندومي؟** | **81.** |
|  | **4-يوميا** | **3- من 2 الى 5 مرات اسبوعيا** | **2-مرة اسبوعيا** | **1-لا** |  |
|  |  |  |  | **هل يأكل الطفل الشكولاتة او الحلويات السكرية؟** | **82.** |
|  | **4-يوميا** | **3- من2 الى 5 مرات اسبوعيا** | **-2- مرة اسبوعيا** | **1-لا** |  |
