# Supplementary material for: Association between some environmental risk factors and attention-deficit hyperactivity disorder among children in Egypt: a case-control study
Source: Ital J Pediatr. 2025 Jan 29;51:19. doi: 10.1186/s13052-025-01843-w (PMC11776284; doi:10.1186/s13052-025-01843-w)
Supplement: Supplementary file 2 — Supplementary Material 2 [file 13052_2025_1843_MOESM2_ESM.docx]

**Supplementary file Tables**

**Association between some environmental risk factors and attention-deficit hyperactivity disorder among children in Egypt: A case-control study**

**Table (1): Estimation for risk of development of ADHD in children in relation to sociodemographic characteristics, parental occupation, the history of mother’s gestational period, the neonatal health status, and parental smoking at home (Pediatric Alexandria University Hospital, 2022).**

| **Risk Factors** | | **Cases**  **(n = 126)** | **Controls**  **(n = 126)** | **Test of Significance**  **(p-value)** |
| --- | --- | --- | --- | --- |
|  |  | **N (%)** | **N (%)** |  |
| **Age of mother at conception of the child under study (years)**  < 20  20-35 (Ref)  35+ | | 8(6.4)  111(88)  7(5.6) | 0 (0)  117 (92.8)  9 (7.2) | χ^2^=8.4 (0.01*) |
| Mean ± SD. | | 28.15±4.6 | 29.33±3.46 | t=2.42 (0.016*) |
| **Age of father at conception of the child under study (years)**  20-39 (Ref)  40+ | | 105 (83.3)  21 (16.7) | 114 (90.3)  12 (9.7) | χ^2^=2.8 (0.09) |
| Mean ± SD. | | 34±5.01 | 33.84±4.64 | t=0.28 (0.78) |
| **Level of education of the mother**  Low  Intermediate  High (Ref) | | 66 (52.4)  27 (21.4)  33 (26.2) | 15 (11.9)  15 (11.9)  96 (76.2) | χ^2^=66.30 (<0.001*) |
| **Level of education of the father**  Low  Intermediate  High (Ref) | | 61 (48.4)  32 (25.4)  33 (26.2) | 14 (11.1)  15 (11.9)  97 (77) | χ^2^=66.30 (<0.001*) |
| **Crowding index**  <1(Ref)  1-  **2+** | | 16 (12.7)  89 (69.1)  21 (18.2) | 30 (23.8)  91 (72.3)  5 (3.9) | χ^2^=14.1 (<0.001*) |
| **Parental consanguinity**  No (Ref)  Yes | | 105 (83.3)  21 (16.7) | 123 (97.6)  3 (2.4) | χ^2^=14.86 (0.001*) |
| **Mother occupation**  Housewives (Ref)  Working | 104 (82.5)  22 (17.5) | 96 (76.2)  30 (23.8) | χ^2^=1.55 (0.213) |  |
|  |  |  |  |  |
| **Father occupation**  No work (Ref)  Working | 3 (2.4)  123 (97.6) | 2 (1.6)  124 (98.4) | χ^2^=0.20 (1.00 ^FE^) |  |
| **Exposure of the father to physical risk factors**  No (Ref)  Yes | 120 (95.2)  6 (4.8) | 125 (99.2)  1 (0.8) | χ^2^=3.673 (0.12 ^FE^) |  |
| **Exposure of the father to biological risk factors**  No (Ref)  Yes | 118 (93.7)  8 (6.3) | 123 (97.6)  3 (2.4) | χ^2^=2.376 (0.123) |  |
| **Exposure of the father to chemical risk factors**  No (Ref)  Yes | 77 (61.1)  49 (38.9) | 123 (97.6)  3 (2.4) | χ^2^=51.27 (<0.001*) |  |
| **Spacing between the 2 pregnancies in months**  Min. – Max.  Median (IQR) | **(n = 73)**  2.0 – 63.0  4.0 (3.0–7.0) | **(n = 66)**  3.0 – 108.0  36.0 (24.0–60.0) | U=684.50 (<0.001*) |  |
| **Vitamins intake**  No  Yes (Ref) | | 19 (15.1)  107 (84.9) | 3 (2.4)  123 (97.6) | χ^2^=12.69 (<0.001*) |
| **Delivery mode**  Normal vaginal delivery (Ref)  Caesarian Section | | 62 (49.2)  64 (50.8) | 38 (30.2)  88 (69.8) | χ^2^=9.55 (0.002*) |
| **Weight of the child at birth**  Normal weight 2500 gram (Ref)  Weight less than 2500 gram | | 68 (38.1)  58 (20.6) | 96 (76.2)  30 (23.8) | χ^2^=13.6 (<0.001*) |
| **Suffering of the child from any disease**  No diseases (Ref)  Neonatal jaundice for long period  Asthma | | 74 (58.8)  42 (33.3)  10 (7.9) | 113 (89.7)  12 (9.5)  1 (0.8) | χ^2^=32.16 (<0.0001*) |
| **Type of Feeding**  Bottle feeding  Breast feeding (Ref) | | 10 (7.9)  116 (92.1) | 3 (2.4)  123 (97.6) | χ^2^=3.97 (0.046*) |
| **Tobacco smoking of the mother**  No (Ref)  Yes | | 90 (71.4)  36 (28.6) | 107 (84.9)  19 (15.1) | χ^2^=6.72 (0.01*) |
| **Tobacco smoking of the father**  No (Ref)  Yes | | 62 (49.2)  64 (50.8) | 66 (52.4)  60 (47.6) | χ^2^=0.25 (0.61) |

Ref is reference, χ^2^: Chi square test, FE**:** Fisher Exact test, t: Student t-test, U: Mann Whitney test, *: Statistically significant at p < 0.05.

**Table (2):** **Risk estimation of ADHD regarding some maternal habits, maternal exposure to electromagnetic radiation, and exposure to some indoor and outdoor environmental factors (Pediatric Alexandria University Hospital, 2022).**

| **Risk Factors** | **Case (n = 126)** | **Control**  **(n = 126)** | **Test of significance**  **(p-value)** |
| --- | --- | --- | --- |
|  | **No. (%)** | **No. (%)** |  |
| **Using of newspaper to wrap food**  No (Ref)  1-2 times weekly  ≥3 times weekly | 48 (38.1)  28 (22.2)  50 (39.7) | 118 93.7)  6 (4.8)  2 (1.5) | χ^2^=88.06  (<0.001*) |
| **Using Aluminum utensils in cooking**  Once or twice per week (Ref)  ≥3 times weekly | 3 (2.4)  123 (97.6) | 20 (15.9)  106 (84.1) | χ^2^=13.83 (<0.001*) |
|  |  |  |  |
|  |  |  |  |
| **Using of unpackaged flour**  No (Ref)  1-2 times weekly  ≥3 times weekly | 46 (36.5)  52 (41.3)  28 (22.2) | 122 (96.8)  3 (2.4)  1 (0.8) | χ^2^=103.17 (0.001*) |
| **Daily usage of mobile phone**  **during pregnancy period**  No (Ref)  <10 minutes  10–30 minutes  > 30 minutes | 8 (6.3)  55 (43.7)  59 (46.8)  4 (3.2) | 3 (2.4)  61 (48.4)  58 (46.0)  4 (3.2) | χ^2^=2.59 (0.459) |
| **Maternal daily usage of computer during pregnancy period**  No (Ref)  <2 hours daily  2–5 hours daily  > 5 hours | 96 (76.2)  15 (11.9)  11 (8.7)  4 (3.2) | 8 (6.3)  70 (55.6)  9 (7.1)  39 (31.0) | χ^2^=138.74 (0.001*) |
| **Maternal daily hours of TV watching during pregnancy period**  No (Ref)  <2 hours  2–5 hours  > 5 hours | 2 (1.6)  41 (32.5)  52 (41.3)  31 (24.6) | 1 (0.8)  61 (48.4)  56 (44.4)  8 (6.3) | χ^2^= 17.97 (<0.001*) |
| **Using of pesticides at home**  No (Ref)  Sometimes  ≥3 times weekly | 2 (1.6)  75 (59.5)  49 (38.9) | 8 (6.3)  100 (79.4)  18 (14.3) | χ^2^=21.52 (<0.001*) |
| **Source of water at home**  Water company (Ref)  Groundwater untreated | 114 (90.5)  12 (9.5) | 124 (98.4)  2 (1.6) | χ^2^=7.56 (0.005*) |
| **Type of pipes of water at home**  Old leaded pipes  New plastic pipes (Ref) | 62 (49.2)  64 (50.8) | 33 (26.2)  93 (73.8) | χ^2^=14.21 (<0.001*) |
| **Type of fuel used at home**  Natural gas (Ref)  Others | 21 (16.7)  105 (83.3) | 113 (89.7)  13 (10.3) | χ^2^=134.89 (<0.001*) |
| **Sufficient natural lighting and aeration at home**  No  Yes (Ref) | 77 (61.1)  49 (38.9) | 48 (38.1)  78 (61.9) | χ^2^=13.35 (<0.001*) |
| **Type of painted surfaces**  Wallpaper (Ref)  Old oil paint  New oil paint  Plastic paint | 25 (19.8  92 (73.0)  3 (2.4)  6 (4.8) | 18 (14.3)  9 (7.1)  29 (23.0)  70 (55.6) | χ^2^=144.37 (<0.001*) |
| **Living near highway**  No (Ref)  Yes | 63 (50.0)  63 (50.0) | 123 (97.6)  3 (2.4) | χ^2^=73.90  (<0.001*) |
| **Living near high traffic street**  No (Ref)  Yes | 62 (49.2)  64 (50.8) | 82 (65.0)  44 (35.0) | χ^2^= 6.48  (0.01) |
| **Living near gas station**  No (Ref)  Yes | 56 (44.4)  70 (55.6) | 120 (95.2)  6 (4.8) | χ^2^= 77.17 (<0.001*) |
| **Living near landfill**  No (Ref)  Yes | 115 (91.3)  11 (8.7) | 125 (99.2)  1 (0.8) | χ^2^= 8.75 (0.003) |
| **Living near industrial area**  No (Ref)  Yes | 107 (84.9)  19 (15.1) | 117 (92.9)  9 (7.1) | χ^2^= 4.02 (0.045) |
| **Living near agriculture area**  No (Ref)  Yes | 72 (57.1)  54 (42.9) | 124 (98.4)  2 (1.6) | χ^2^=62.08 (<0.001*) |

Ref is reference, χ^2^: Chi square test, *: Statistically significant at p < 0.05.

**Table (3):** **Risk of ADHD in relation to lifestyle of the child, exposure to electromagnetic radiation, and dietary and snacking habits (Pediatric Alexandria University Hospital, 2022).**

| **Risk factors** | **Case  (n = 126)** | | **Control  (n = 126)** | **Test of significance**  **(p-value)** |
| --- | --- | --- | --- | --- |
|  | **No. (%)** | | **No. (%).** |  |
| **Child plays with plastic toys**  No (Ref)  Yes | 2 (1.6)  124 (98.4) | | 16 (12.7)  110 (87.3) | χ^2^=11.73 (0.001*) |
| **Exposure of the child to household pesticides**  No (Ref)  Yes | 11 (8.7)  115 (91.3) | | 108 (85.7)  18 (14.3) | χ^2^=149.81 (<0.001*) |
| **The child’s daily watching of TV**  No (Ref)  <2 hours  2–5 hours  > 5 hours | 1 (0.8)  16 (12.7)  44 (34.9)  65 (51.6) | | 4 (3.2)  52 (41.3)  62 (49.2)  8 (6.3) | χ^2^=74.26 (<0.001* ^MC^) |
| **The child’s daily usage of mobile phone**  ≤2 hours/ day (Ref)  >2 hours/ day | 14 (11.1)  112 (88.9) | | 31 (24.6)  95 (75.4) | χ^2^=7.82 (0.005*) |
| **The child’s daily usage of computer**  ≤2 hours/ day (Ref)  >2 hours/ day | 19 (15.1)  107 (84.9) | | 35 (27.8)  91 (72.2) | χ^2^=6.03 (0.014*) |
| **Eating potato crisps**  No (Ref)  Once weekly  2–5 times weekly | 13 (10.3)  18 (14.3)  95 (75.4) | | 63 (50.0)  46 (36.5)  17 (13.5) | χ^2^=99.47 (<0.001*) |
| **Eating commercial packed noodles**  No (Ref)  Once a week  2–5 times weekly  Daily | 6 (4.8)  39 (31.0)  71 (56.3)  10 (7.9) | | 71 (56.3)  27 (21.4)  25 (19.8)  3 (2.4) | χ^2^=82.86 (<0.001*) |
| **Drinking of milk**  No  Once a week  2–5 times weekly  Daily (Ref) | 35 (27.8)  43 (34.1)  30 (23.8)  18 (14.3) | | 4 (3.2)  11 (8.7)  66 (52.4)  45 (35.7) | χ^2^=68.68 (<0.001*) |
| **Eating vegetable and fruits**  No (Ref)  ≤3 times weekly  Daily | 87 (69.0)  2 (1.6)  37 (29.4) | | 40 (31.8)  8 (6.3)  78 (61.9) | χ^2^=35.61 (<0.001*) |
| **Drinking of carbonated beverages**  No (Ref)  Once per week  2–5 times weekly  Daily | 20 (15.9)  57 (45.2)  47 (37.3)  2 (1.6) | | 48 (38.1)  46 (36.5)  29 (23.0)  3 (2.4) | χ^2^=17.39*  (0.001* ^MC^) |
| **Eating sweets**  No (Ref)  2–5 times weekly  Once weekly  Daily | | 0 (0.0)  33 (26.2)  18 (14.3)  75 (59.5) | 2 (1.6)  45 (35.7)  34 (27.0)  45 (35.7) | χ^2^=15.9 (<0.001*) |

Ref: reference, χ2: Chi square test, MC: Monte Carlo test, *: Statistically significant at p ≤ 0.05
