# Supplementary material for: Association between some environmental risk factors and attention-deficit hyperactivity disorder among children in Egypt: a case-control study
Source: Ital J Pediatr. 2025 Jan 29;51:19. doi: 10.1186/s13052-025-01843-w (PMC11776284; doi:10.1186/s13052-025-01843-w)
Supplement: Supplementary file 4 — Supplementary Material 4 [file 13052_2025_1843_MOESM4_ESM.docx]

**Cover letter**

[Rania Hussein Refai]
[Doctor of Public Health Sciences, Alexandria University]
[Alexandria, Egypt]

Dear,

We wish to submit an original research article entitled “**Association between some environmental risk factors and attention-deficit hyperactivity disorder among children in Egypt: A case-control study**” for consideration by [*Italian Journal of Pediatrics*].

We confirm that this work is original and has not been published elsewhere, nor is it currently under consideration for publication elsewhere. Also, there was no prior discussion with your editorial board member about the work described in this manuscript. The publication of this manuscript has been approved by all co-authors.

In this paper, we reported some significant environmental exposures that may be associated with the risk of Attention-Deficit Hyperactivity Disorder (ADHD). This adds evidence that ADHD can be environmentally induced. Recognizing these factors that trigger the disease onset and progression would help to take measures to reduce the individuals’ risk.

We believe that this manuscript is appropriate for publication by [*Italian Journal of Pediatrics*] because it assesses some environmental exposures in relation to ADHD which are crucial in improving the plan of the management of the ADHD children.

Patient samples were chosen based on an accurate diagnosis without any prior selection thus the samples appeared to be representative of the population.

We have no conflicts of interest to disclose. In addition, we confirm that all authors have approved the manuscript for submission.

Please address all correspondence concerning this manuscript to me at [hiph.rattia@alexu.edu.eg].

Thank you for your consideration of this manuscript.

Sincerely,

Rania
