# Supplementary material for: Association between some environmental risk factors and attention-deficit hyperactivity disorder among children in Egypt: a case-control study
Source: Ital J Pediatr. 2025 Jan 29;51:19. doi: 10.1186/s13052-025-01843-w (PMC11776284; doi:10.1186/s13052-025-01843-w)
Supplement: Supplementary file 3 — Supplementary Material 3 [file 13052_2025_1843_MOESM3_ESM.docx]

**Point by point response to reviewers’ comments**

**Authors: We are grateful for your careful review of our manuscript and your valuable feedback. Your suggestions have been instrumental in strengthening our study. We have carefully incorporated your recommendations into the revised version of our manuscript.**

Reviewer #2:

The manuscript has a potential value but needs to be improved in the macrostructure and on some its parts:

**Authors: The structure of the manuscript was revised and improved and all comments were properly discussed.**

Reviewer #2:

In the introduction the Authors should emphasize the early diagnosis of neuropsychiatric syndromes (see SARS-CoV-2 related paediatric acute-onset neuropsychiatric syndrome Lancet Child Adolesc Health 2021) and the key role of early and prompt diagnosis of attention-deficit hyperactivity disorders and the importance for a pediatricians to a "true" its prevention:

**Authors: The significance of early diagnosis and intervention of ADHD was mentioned along with the citation references: page 5; lines 97-106.**

Reviewer #2:

The manuscript goals should be better enunciated in the final part of the introduction:

**Authors: The goal of the study was articulated to the final part of the introduction: page 5; lines 107-110.**

Reviewer #2:

Methods

Please cite STROBE Statement, a guidelines for reporting observational studies to well report the case reports:

**Authors: The manuscript was carefully checked using (STROBE) guidelines for case-control studies to ensure that all details needed were incorporated in the manuscript: pages 5-6; lines 114-117.**

**The completed checklist is attached at the end of this file.**

Reviewer #2:

Results

Please re organize the figures current it is difficult to follow:

**Authors: Thank you for your valuable feedback. We have carefully reviewed your comments regarding the organization of the figures (which we believe you meant to refer to as tables). To improve the clarity and readability of our findings, we have made the following changes:**

- **Removed unnecessary columns: We have removed the column of test of significance (Chi-squared test, t-test, and Mann Whitney tests) and we removed the p value column of the odds ratio from Tables 1-3. The confidence intervals provided in these tables are sufficient to indicate the statistical significance of the odds ratios without the need for repetition by p value. We put the results of Chi-squared test, t-test, and Mann Whitney test in the supplementary file tables.**
- **Simplified tables: We have divided Tables 1 and 2 into smaller, more focused tables to reduce their length and improve their readability. We have divided table 1 into two tables (Table 1 (Estimation for risk of development of ADHD in children in relation to socio-demographic characteristics, parental occupation, and parental smoking at home) and Table 2 (Estimation for risk of development of ADHD in children in relation to the history of mother’s gestational period and the infant health status)). We have also divided table 2 into two tables (Table 3 (Risk estimation of ADHD regarding some maternal habits and maternal exposure to electromagnetic radiation) and Table 4 (Risk estimation of ADHD regarding exposure to some indoor and outdoor environmental factors))**
- **Reorganized regression analysis table: We have removed the Coefficient B column from the regression analysis Table (now became Table 7), as the adjusted odds ratios provide a clear indication of the magnitude of the risk associated with each factor without the need for the repetition through Coefficient B values.**

**We think these changes could make it easier for readers to understand and interpret our findings.**

Reviewer #2:

This case-control study is shedding light on the most important risk factors related to ADHD

Please to be more precise on exposure to chemical agents during the father's work yields a higher odds ratio regarding the offspring's ADHD

**Authors: Thank you for your valuable feedback. To address your concerns, we have revised and rephrased the paragraph to focus solely on the significant exposure identified in our analysis: exposure of the father to chemical agents (petroleum products, paints, or organic solvents) during work: page 12; lines 263-265.**

Reviewer #2:

the conclusions should be shorter and less absolutes on the statements:

**Authors: The conclusion was adjusted as requested: page 28; lines 573-578.**

**STROBE Statement checklist**

|  | **Item No.** | **Recommendation** | **Page  No.** | **Relevant text from manuscript** |
| --- | --- | --- | --- | --- |
| **Title and abstract** | 1 | (*a*) Indicate the study’s design with a commonly used term in the title or the abstract | P.1 | Title |
|  |  | (*b*) Provide in the abstract an informative and balanced summary of what was done and what was found | P.2 | Abstract |
| **Introduction** | | | |  |
| Background/rationale | 2 | Explain the scientific background and rationale for the investigation being reported | P.3-5 | Background |
| Objectives | 3 | State specific objectives, including any prespecified hypotheses | P.5 | Last paragraph of background |
| **Methods** | | | |  |
| Study design | 4 | Present key elements of study design early in the paper | P.5 | Methods: line 113 |
| Setting | 5 | Describe the setting, locations, and relevant dates, including periods of recruitment, exposure, follow-up, and data collection | P.5 | Methods: Lines 113, 114 |
| Participants | 6 | (a) Give the eligibility criteria, and the sources and methods of case ascertainment and control selection. Give the rationale for the choice of cases and controls  (b) For matched studies, give matching criteria and the number of controls per case | P.6 | Target population: lines 118-124 |
| Variables | 7 | Clearly define all outcomes, exposures, predictors, potential confounders, and effect modifiers. | P. 6-7 | Variables: lines 131-147 |
| Data sources/ measurement | 8* | For each variable of interest, give sources of data and details of methods of assessment (measurement). Describe comparability of assessment methods if there is more than one group | P. 7-9 | Data collection methods and tools |
| Bias | 9 | Describe any efforts to address potential sources of bias | P. 6 and P. 20 | inclusion & exclusion criteria and statistically by regression analysis |
| Study size | 10 | Explain how the study size was arrived at | P.6 | Sample size: Lines 125-130 |

STROBE Statement—checklist of items that should be included in reports of ***case-control studies*** for the manuscript entitled “Association between some environmental risk factors and attention-deficit hyperactivity disorder among children in Egypt: A case-control study".

| Quantitative variables | 11 | Explain how quantitative variables were handled in the analyses. If applicable, describe which groupings were chosen and why | P.10-11 | Statistical design and analysis: lines 224-240 |
| --- | --- | --- | --- | --- |
| Statistical methods | 12 | (a) Describe all statistical methods, including those used to control for confounding | P.10-11 | Statistical design and analysis: lines 224-240 |
|  |  | (b) Describe any methods used to examine subgroups and interactions | P.10-11 | Statistical design and analysis: lines 224-240 |
|  |  | (c) Explain how missing data were addressed | No missing data |  |
|  |  | (d) If applicable, explain how matching of cases and controls was addressed | P.6 | Target population: Line 122 |
|  |  | (e) Describe any sensitivity analyses | P.11 | Statistical design and analysis: Regression analysis: lines 238-240 |
| **Results** | | | | |
| Participants | 13* | (a) Report numbers of individuals at each stage of study—eg numbers potentially eligible, examined for eligibility, confirmed eligible, included in the study, completing follow-up, and analysed | P.11 | 1^st^ paragraph of the results section |
|  |  | (b) Give reasons for non-participation at each stage |  | Not applicable |
|  |  | (c) Consider use of a flow diagram |  | --- |
| Descriptive data | 14* | (a) Give characteristics of study participants (eg demographic, clinical, social) and information on exposures and potential confounders | P. 12-13 | Distribution of the study sample Table 1 |
|  |  | (b) Indicate number of participants with missing data for each variable of interest |  | Not applicable |
| Outcome data | 15* | Report numbers of outcome events or summary measures | P. 12-21 | Tables 1-7 |
| Main results | 16 | (*a*) Give unadjusted estimates and, if applicable, confounder-adjusted estimates and their precision (eg, 95% confidence interval). Make clear which confounders were adjusted for and why they were included | P. 12-21 | Tables 1-7 |
|  |  | (*b*) Report category boundaries when continuous variables were categorized | P. 20-21 | P< 0.05 (Tables 6, 7) |
|  |  |  |  |  |

| Other analyses | 17 | Report other analyses done—eg analyses of subgroups and interactions, and sensitivity analyses |  | --- |
| --- | --- | --- | --- | --- |
| **Discussion** | | | | |
| Key results | 18 | Summarise key results with reference to study objectives | P.21 | First paragraph: lines 399-403 |
| Limitations | 19 | Discuss limitations of the study, taking into account sources of potential bias or imprecision. Discuss both direction and magnitude of any potential bias | P. 28 | Limitations: lines 557-564 |
| Interpretation | 20 | Give a cautious overall interpretation of results considering objectives, limitations, multiplicity of analyses, results from similar studies, and other relevant evidence | P.21-28 | Discussion, limitations and strengths, conclusion |
| Generalisability | 21 | Discuss the generalisability (external validity) of the study results | P.28 | Points of strength of the study: lines 565-571 |
| **Other information** | |  | | |
| Funding | 22 | Give the source of funding and the role of the funders for the present study and, if applicable, for the original study on which the present article is based | P. 30 | Self-funded: Lines 614-615 |

**Thank you**
